# Persistent hyperparathyroidism after kidney transplantation in children

**DOI:** 10.1080/0886022X.2025.2511279

**Published:** 2025-06-01

**Authors:** Peong Gang Park, Ahram Han, Yo Han Ahn, Sangil Min, Jongwon Ha, Hee Gyung Kang, Hyun Kyung Lee

**Affiliations:** ^a^ Departments of Pediatrics, Ajou University School of Medicine, Suwon, Republic of Korea; ^b^ Department of Surgery, Seoul National University College of Medicine, Seoul, Korea; ^c^ Transplantation Research Institute, Seoul National University Medical Research Center, Seoul, Korea; ^d^ Departments of Pediatrics, Seoul National University College of Medicine, Seoul National University Children’s Hospital, Seoul, Republic of Korea; ^e^ Kidney Research Institute, Seoul National University Medical Research Center, Seoul, Korea; ^f^ Department of Pediatrics, Chung-Ang University College of Medicine, Chung-Ang University Hospital, Seoul, Korea

**Keywords:** Kidney transplantation, persistent hyperparathyroidism, kidney failure, graft outcome

## Abstract

**Background:**

Persistent hyperparathyroidism after kidney transplantation (KT) has been reported in up to 50% of adult recipients, but pediatric data remain limited. We evaluated the prevalence, skeletal manifestations, and risk factors for persistent hyperparathyroidism in children following KT.

**Methods:**

In this retrospective cohort study, 107 pediatric KT recipients (58% male; median age 10.3 years) transplanted between 2004 and 2019 were analyzed. Persistent hyperparathyroidism was defined as a median parathyroid hormone (PTH) > 65 pg/mL between 3 and 12 months post-KT. Risk factors for persistent hyperparathyroidism, post KT clinical features, and treatment status were analyzed.

**Results:**

Thirty-six patients (33.6%) had persistent hyperparathyroidism after KT. On univariable analysis, dialysis duration of 24 months or longer (*p* = 0.028) and pretransplant hyperphosphatemia (*p* = 0.026) were significantly associated with persistent hyperparathyroidism. The multivariable model identified pretransplant hyperphosphatemia as an independent predictor (OR 2.70, 95% CI 1.10–6.87; *p* = 0.030). There was no significant difference in height Z score change between patients with and without persistent hyperparathyroidism (*p* = 0.97). However, persistent hyperparathyroidism was associated with poorer graft survival (log-rank *p* = 0.049). Six patients received cinacalcet and one underwent subtotal parathyroidectomy for refractory hypercalcemia.

**Conclusions:**

Persistent hyperparathyroidism is relatively common in pediatric KT recipients, affecting one-third of patients by one-year post-transplant. Prolonged dialysis and pre-existing hyperphosphatemia before KT may be risk factors. These findings underscore the importance of optimizing chronic kidney disease-mineral bone disease management and routine PTH monitoring before and after transplant in children.

## Introduction

Secondary hyperparathyroidism is a common manifestation of chronic kidney disease-mineral bone disease (CKD-MBD). CKD-MBD is a multifaceted complication experienced not only by patients on dialysis but also those in the early stages of chronic kidney disease (CKD) [1]. In response to declining kidney function, fibroblast growth factor 23 (FGF-23) production increases to induce phosphaturia in order to maintain normal phosphate levels, thereby reducing active vitamin D synthesis, leading to reduced calcium absorption, secondary hyperparathyroidism, and increased bone resorption, which ultimately results in CKD-MBD [2–4]. Following kidney transplantation (KT), parathyroid hormone (PTH) secretion normalizes as the glomerular filtration and endocrine function of the kidney are restored [5]. However, studies in adults indicate that persistent hyperparathyroidism can remain even after eGFR normalizes, with prevalence rates reported between 15% and 50% from 3 months to 5 years after KT [6–11]. The suggested reasons include persistence of structural changes such as parathyroid gland hyperplasia and calcium-sensing receptor/vitamin D receptor downregulation even after KT.

Unlike secondary hyperparathyroidism in CKD, hyperparathyroidism after KT is accompanied by hypophosphatemia or hypercalcemia, which is similar to primary hyperparathyroidism [6]. In addition, persistent hyperparathyroidism in KT recipients has been linked to various complications such as fracture, urinary stones, decreased graft function, ectopic calcification, cardiovascular events, and mortality [12–14]. Suggested risk factors of persistent hyperparathyroidism include dialysis vintage [15,16], high pretransplant serum phosphorus [6,15], and high pretransplant PTH [17,18]. Several reviews recommend proactive treatments such as calcimimetics or parathyroidectomy if hypercalcemia accompanies post-KT hyperparathyroidism. Conversely, some recommend vigilant monitoring including active identification and management of vitamin D deficiency on the basis that mild to moderate increases in PTH levels in long-term kidney transplant recipients may not be linked with bone loss or fractures [19–22].

As the major causes of CKD and the characteristics of kidney failure differ between children and adults, the epidemiology of persistent hyperparathyroidism in children is expected to differ from that in adults. In addition, persistent hyperparathyroidism might have an adverse effect on linear growth, giving it more significant clinical implication in children [23]. However, research focusing on hyperparathyroidism post-KT in the pediatric population remains scarce. Available studies indicate a prevalence rate of approximately 40%, identifying dialysis duration prior to transplantation as a major risk factor, consistent with observations in adult cohorts [24]. In Korea, one study on 46 children have found a 26% prevalence of hyperparathyroidism; key risk factors identified include prolonged dialysis and elevated PTH levels before and after KT [23].

Given the significance of persistent hyperparathyroidism and its under-researched status in pediatric patients, we assessed the prevalence and associated clinical features of persistent hyperparathyroidism post-KT in Korean pediatric KT patients.

## Methods

### Data source and participant selection

We retrospectively reviewed the medical records of pediatric patients at Seoul National University Children’s Hospital who underwent KT between January 2004 to December 2019. Eligible subjects were children under 18 years of age at the time of KT who had their parathyroid function checked pretransplant and at least one PTH measurement between 3 months and 1-year post-transplant. We collected medical histories including the causes of kidney failure, dialysis modality, and duration prior to transplantation, and graft failure. All longitudinal data, including laboratory results such as the levels of PTH, calcium, phosphorus, vitamin D, and creatinine, alkaline phosphatase, immunosuppressant prescriptions, and operation history, were collected through clinical data warehouse. Any data recorded after graft failure or parathyroidectomy were excluded from analysis. The study was approved by the Seoul National University Hospital institutional review board (IRB No. 2106-123-1228). The need for informed consent from the patients was waived due to the retrospective nature of the study. All statistical analyses were performed using R-project version 4.2.1 (R core team, Vienna, Austria).

### Descriptive data analysis

The definition of post-KT hyperparathyroidism has not been firmly established. However, many studies define it based on stabilized values several months after transplantation. Therefore, based on previous pediatric studies and reports from some adult studies, we defined post-KT hyperparathyroidism as a median PTH level greater than 65 pg/mL between 3 months and 1-year post-transplant [2,11,24,25]. Hypercalcemia and hypocalcemia, as well as hyperphosphatemia and hypophosphatemia, were defined as median calcium and phosphorus levels above the upper limit or below the lower limit according to the child’s age, respectively (Supplementary Table 1) [8,26]. Pretransplant hyperphosphatemia was defined as a serum phosphate level more than the target level for kidney failure according to their age. The causes of kidney failure were categorized as congenital anomalies of kidney and urinary tract (CAKUT), glomerulopathy, and other causes of kidney failure. The continuous variables are presented as median and interquartile ranges, and categorical variables are presented as numbers and percentages. Considering the nonnormal distribution of data, statistical comparisons were performed using either the Wilcoxon rank sum test or the chi-squared test, with an alpha value of 0.05.

### Multivariable data analysis

To assess the association between post-KT hyperparathyroidism and clinical features, multivariable logistic regression models were made. Covariates selected for inclusion in the models, based on their clinical relevance, included sex, age, cause of kidney failure, dialysis duration, pretransplant hyperparathyroidism, pretransplant hyperphosphatemia, calcium level, immunosuppressant type, and acute rejection.

## Results

### Prevalence of hyperparathyroidism

**Figure 1.**
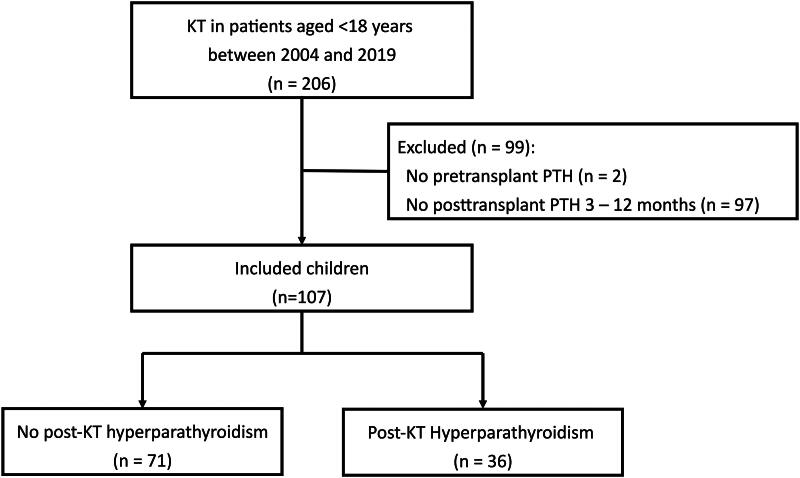
Study flow chart.

From January 2004 to December 2019, a total of 206 children below the age of 18 years underwent KT. Among them, 107 were analyzed for the assessment of hyperparathyroidism status (Figure 1). The median age of these children at KT was 10.6 years (IQR, 7.1 − 13.7), with 62 (57.8%) being male (Table 1). Majority of them received initial immunosuppression with triple therapy of tacrolimus, mycophenolate mofetil, and corticosteroid (*n* = 82, 76.6%).

**Table 1.** Baseline patient characteristics.

Baseline characteristics	Patient cohort (*N* = 107)
Age at KT, yr	10.3 (6.9–13.3)
Male sex, *n*	62 (57.9%)
Height at KT, cm	128.0 (108.0–146.6)
Z score	−1.8 (-2.6–1.1)
Weight at KT, kg	27.1 (17.3–36.8)
BMI at KT, kg/m^2^	16.1 (14.9–18.0)
Cause of kidney failure, *n*	
CAKUT	53 (50.0%)
GN	21 (19.8%)
Other	32 (30.2%)
Preemptive KT, n	20 (18.7%)
Immunosuppressive regimen	
Tacrolimus, mycophenolate, prednisolone	82 (76.6%)
Cyclosporine, mycophenolate, prednisolone	22 (20.6%)
Other	3 (2.8%)
Dialysis duration, month	21 (1.5–40)
Last laboratory findings	
Parathyroid hormone (pg/mL)	181.1 (100.8–327.8)
Calcium (mg/dL)	9.7 (9.4–10.0)
Phosphorus (mg/dL)	5.6 (4.8–6.4)
Alkaline phosphatase (IU/L)	256 (153–336)
Donor type	
Deceased	43 (40.2%)
Living-related	64 (59.8%)
Post KT parathyroid hormone (pg/mL)	48.0 (33.0–74.0)

Abbreviations: KT, kidney transplantation; CAKUT, congenital anomaly of kidney and urinary tract; GN, glomerulonephritis.

The median PTH level was 181.1 pg/mL (IQR, 100.8–327.8) before KT, which subsequently decreased to 48.0 pg/mL (IQR, 33.0–74.0) after transplantation. However, 36 of 107 children (33.4%) had persistent hyperparathyroidism between 3 and 12 months post-transplant, and among these 36, 13 underwent testing at 2 years; 11 of them continued to show elevated PTH. Additionally, of the 36 children with hyperparathyroidism at one-year post-KT, seven had hypercalcemia and three had hypophosphatemia.

### Association of demographic and pretransplant clinical features and hyperparathyroidism one-year post-KT

Children with hyperparathyroidism one-year post-KT were more likely to have undergone prolonged dialysis (*p* = 0.028) and had pretransplant hyperphosphatemia (*p* = 0.026) (Table 2). No significant differences were found in pretransplant calcium or ALP levels. In multivariable analysis, pretransplant hyperphosphatemia was significantly associated with posttransplant hyperparathyroidism (OR 2.70, 95% CI 1.10–6.87) (Table 3).

**Table 2.** Comparison of demographic and clinical feature between children with and without posttransplant hyperparathyroidism.

	No hyperparathyroidism (*N* = 71)	Hyperparathyroidism (*N* = 36)	*P* Value
Age at KT (years)	9.8 (7.5–13.4)	10.3 (5.6–13.2)	0.363
Male sex	41 (57.7%)	21 (58.3%)	1.000
Cause of Kidney failure			0.501
CAKUT	33 (46.5%)	20 (57.1%)	
GN	16 (22.5%)	5 (14.3%)	
Other	22 (31.0%)	10 (28.6%)	
Preemptive KT	13 (18.3%)	7 (19.4%)	>0.999
Dialysis vintage (months)	14 (1.5–33.5)	30 (2–48)	0.072
Dialysis more than 24 months	26 (36.6%)	22 (61.1%)	0.028
Pretransplant laboratory findings			
Parathyroid hormone (pg/mL)	171.0 (100.0–340.0)	189.6 (111.9 − 421.5)	0.769
25-OH Vitamin D (ng/mL)	21.4 (13.6–28.4)	17.5 (11.6–22.9)	0.133
Calcium (mg/dL)	9.6 (9.2–9.9)	9.8 (9.5–10.1)	0.140
Phosphorus (mg/dL)	5.6 (4.7–6.3)	5.9 (5.3–6.8)	0.033
Alkaline phosphatase (IU/L)	261 (153–327)	240 (162–354)	0.779
Pretransplant hyperphosphatemia	24 (33.8%)	21 (58.3%)	0.026
Donor type			0.727
Deceased donor	28 (39.4%)	15 (41.7%)	
Living donor	43 (60.6%)	21 (58.3%)	
Acute rejection	20 (28.2)	8 (22.9)	0.727
Height Z score at KT	−1.9 (−2.6−−1.4)	−1.6 (–2.5−−0.8)	0.413
Height Z score change one-year post-KT	−0.8 (−1.4−0.0)	−0.4 (–1.5−−0.6)	0.290
Posttransplant laboratory findings			
Calcium (mg/dL)	9.9 (9.6−10.2)	9.9 (9.6−10.2)	0.520
Alkaline phosphatase (IU/L)	219 (156−283)	201 (150−295)	0.989

Abbreviations: KT, kidney transplantation; PTH, parathyroid hormone.

**Table 3.** Multivariable analysis of associations between one-year posttransplant hyperparathyroidism and demographic and clinical features.

Risk Factor	Adjusted OR	95% CI
Male sex	1.71	0.61−4.80
Age, year	0.96	0.84−1.10
Cause of kidney failure		
CAKUT	Ref.	
Glomerulopathy	0.37	0.10−1.45
Others	0.62	0.21−1.86
Acute rejection	0.65	0.20−2.07
Dialysis duration, months^a^	1.02	1.00−1.04
Pretransplant hyperphosphatemia	2.92	1.12−7.65
Pretransplant Calcium	1.30	0.53−3.18

The outcome examined is the occurrence of one-year post-transplant hyperparathyroidism. The models include the following covariates: sex, age, cause of kidney failure, dialysis duration, pretransplant hyperparathyroidism, pretransplant hyperphosphatemia, calcium level, immunosuppressant type, and acute rejection.

^a^*P* value = 0.10.

Abbreviations: OR, odds ratio; CI, confidence interval; KT, kidney transplantation; CAKUT, congenital anomaly of kidney and urinary tract; GN, glomerulonephritis; PTH, parathyroid hormone.

### Association of hyperparathyroidism with post-KT clinical features

**Figure 2.**
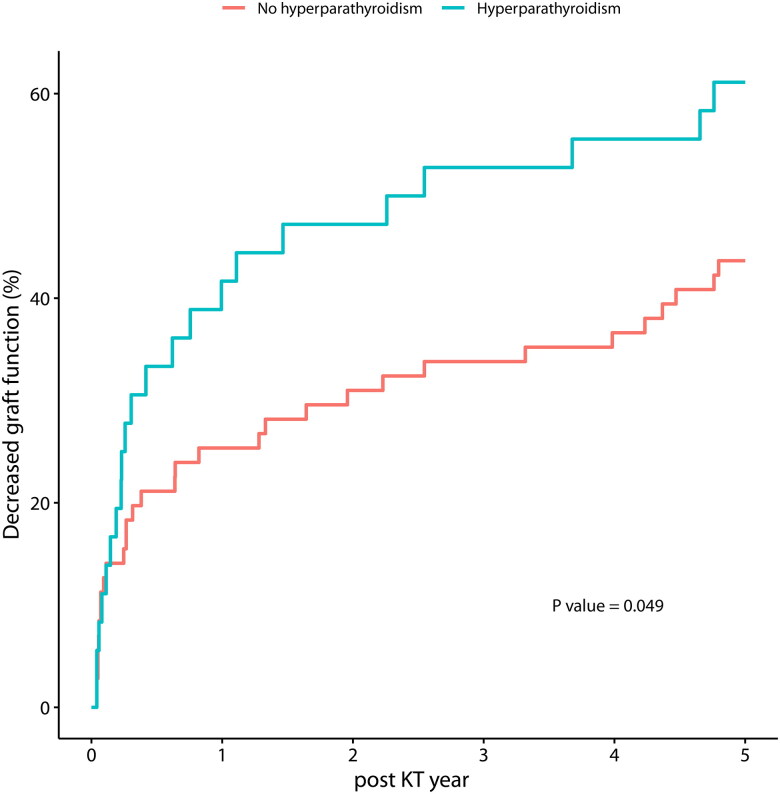
Kaplan–Meier Curve of decreased graft function, according to hyperparathyroidism at one-year post-KT. P-value denotes log-rank test.

The median height Z-score of the children improved by 1.1 (IQR, 0.6–1.8) one year after KT. However, hyperparathyroidism was not associated with changes in height Z-scores at one-year post-KT (median Z-score: 1.1 vs. 1.2, *p* = 0.974). Similarly, no differences in pre- and post-transplant calcium levels were observed between the two groups. A total of 53 out of 107 children (49.6%) either had an incidence of graft failure or an eGFR less than 60 mL/min/1.732 during a median follow-up time of 4.9 years post-KT. Survival analysis indicated that children with posttransplant hyperparathyroidism had worse graft outcomes (log-rank test, *p* = 0.049) (Figure 2).

### Treatment of persistent hyperparathyroidism in children

Of the 36 children with hyperparathyroidism one-year post-KT, 12 received oral phosphorus supplementation due to hypophosphatemia. Six children were treated with cinacalcet, and one underwent surgical intervention. At the onset of cinacalcet therapy, these children had a median serum PTH level of 73.7 pg/mL (IQR, 54.5–110.8) and serum calcium of 10.5 mg/dL (IQR, 10.2–10.6). After a median treatment duration of 15.6 months (IQR, 3.0–19.4 months), hypercalcemia normalized in all but one patient. A 10-year-old boy underwent subtotal parathyroidectomy 24 months post-KT due to persistent hyperparathyroidism and hypercalcemia despite three months of cinacalcet therapy. His serum PTH level was 495 pg/mL and serum calcium was 11.2 mg/dL at the time of surgery. Following parathyroidectomy, his hypercalcemia resolved, and his PTH level decreased to 87.8 pg/mL one-month post-surgery, although it did not fully normalize.

## Discussion

In this study of the Korean single center, about 30% of pediatric KT recipients had persistent hyperparathyroidism despite functioning graft. This is comparable to other studies of children (ranging from 26% to 56%), or adults (ranging from 15% to 65%) [6,12,15,23,24,27,28]. Also, the occurrence of hyperparathyroidism post-KT was associated with decreased graft function, which was also shown in a previous European study, although it is important to acknowledge that various other factors may contribute to this association [24].

As risk factors of persistent hyperparathyroidism, we found dialysis vintage and pretransplant hyperphosphatemia as previously reported in adult and pediatric patients [6,15,16]. Considering that post-KT hyperparathyroidism can arise from structural changes such as parathyroid gland hyperplasia, downregulation of calcium-sensing and vitamin D receptors, and pretransplant alterations in FGF-23 signaling, dialysis vintage may promote parathyroid hyperplasia, while pretransplant hyperphosphatemia may exacerbate CKD-MBD and lead to abnormal FGF-23 signaling. However, our study found no association between persistent hyperparathyroidism and high pretransplant PTH levels or preemptive KT, contrary to previous reports [15,23]. This discrepancy may reflect the limited sample size of our retrospective design, potential differences between pediatric and adult populations, and the absence of detailed pretransplant CKD-MBD management data such as activated vitamin D and phosphate binders.

Both medical and surgical interventions were employed in a cohort of children with hyperparathyroidism and effectively reduced calcium levels, yet their impact on long-term outcomes is uncertain. Actually, previous research on persistent hyperparathyroidism management in children is limited; it is worth noting that treatment of hypercalcemic hyperparathyroidism in adult post-kidney transplant patients has been associated with significant adynamic bone disease [29]. Further studies are needed to determine the best management strategy for children with this condition, weighing the benefits of early treatment against careful monitoring. Of particular note, only a subset of children with persistent hyperparathyroidism exhibited hypercalcemia or hypophosphatemia, and no significant difference in ALP levels were observed. In other words, some patients had isolated elevations in PTH without overt clinical manifestations, raising questions about clinical interpretation. Nonetheless, our findings suggest a possible association between elevated PTH and worse graft outcomes, underscoring the need for vigilant monitoring.

Our findings indicate that hyperparathyroidism following KT is associated with decreased graft function, but due to the retrospective design of our study, causality cannot be established. Even though we excluded values obtained after graft failure, decreased graft function itself may induce secondary hyperparathyroidism through CKD-MBD. Of note, a large-scale European study demonstrated this using a prospective cohort approach, and adult studies that utilized protocol biopsies have shown interstitial calcification in patients with post-KT hyperparathyroidism, which was associated with unfavorable graft outcomes [24,30]. To establish causality, future studies should examine the relationship between parathyroid status and biopsy results in post-KT pediatric patients. Furthermore, although we did not identify a link between hyperparathyroidism and linear growth, higher calcium balance in children suggests the need for further research on their growth patterns, given that proper growth is a key treatment goal in pediatric kidney failure. To date, there is no international guideline pertaining to the surveillance of hyperparathyroidism after KT in children. In our study, many patients did not undergo adequate testing between 3 and 12 months, resulting in a high attrition rate and underscoring the necessity for regular testing. We propose that children receive biannual PTH level screening within the first year after transplant, in accordance with adult protocols, and more frequent screening in cases with risk factors such as longer dialysis duration [31].

This study is limited by its retrospective, single-center design, which may not adequately represent the broader population of pediatric KT recipients. Consequently, selection bias and limited generalizability of the findings should be considered. Due to the lack of a sufficient follow-up period, overall and kidney survival were not fully elucidated, and casual association cannot be identified. Furthermore, although we adjusted for available factors such as rejection status, detailed data on complications (e.g. infections that may affect transplant dysfunction), pretransplant treatments related to CKD-MBD including phosphorus binder use and post-transplant calcium-phosphate management were not available. Despite the identified limitations, this study represents one of the most extensive investigations in Asia examining parathyroid function and calcium-phosphate homeostasis after KT in children. To develop a more comprehensive understanding of the prognosis, future studies necessitate extended follow-up periods and international collaborative efforts.

In conclusion, our study presents comparable prevalence of hyperparathyroidism in children undergoing KT with adults. We also suggest that persistent hyperparathyroidism is related to longer dialysis duration and pretransplant hyperphosphatemia, with substantial patients undergoing treatment. However, establishing proper indication for treatment is necessary. Hence, it’s essential to establish clear treatment guidelines and routinely monitor parathyroid function, especially in children with extended dialysis pre-transplant, as managing hyperparathyroidism could improve graft outcomes.

## Supplementary Material

sup.docx

## Data Availability

Data are available upon reasonable request from the corresponding author.
