# Supplementary material for: Persistent hyperparathyroidism after kidney transplantation in children
Source: Ren Fail. 2025 Jun 1;47(1):2511279. doi: 10.1080/0886022X.2025.2511279 (PMC12128120; doi:10.1080/0886022X.2025.2511279)
Supplement: sup.docx [file IRNF_A_2511279_SM5399.docx]

**Supplementary table 1. Normal value of phosphorus and calcium levels at different ages [1]**

| Variable | Age (years) | Normal value |
| --- | --- | --- |
| Phosphorus (mg/dL) | < 0.5 | 5.2 – 8.4 |
|  | 0.5 – 1 | 5.0 – 7.8 |
|  | 1 –5 | 4.5 – 6.5 |
|  | 6 – 12 | 3.6 – 5.8 |
|  | ≥ 13 | 2.3 – 4.5 |
| Calcium (mg/dL) | ≤ 3 | 8.8 – 11.3 |
|  | 3 – 5 | 9.4 – 10.8 |
|  | 6 – 12 | 9.4 – 10.3 |
|  | ≥ 13 | 8.8 – 10.2 |

Hypercalcemia and hypophosphatemia were defined as median calcium and phosphorus levels above the upper limit or below the lower limit according to the age of the child, respectively.

Reference

[1] KDOQI Work Group (2009) KDOQI Clinical Practice Guideline for Nutrition in Children with CKD: 2008 update. Executive summary. Am J Kidney Dis 53(3 Suppl 2):S11-S104
